# A Cross-Sectional Study to Assess Diabetes as a Risk Factor for Root Caries Amongst the Elderly Population in Udupi District, Karnataka State, India

**DOI:** 10.1155/2024/9963917

**Published:** 2024-07-25

**Authors:** Kush Kalra, Ramprasad Vasthare, Shivashankar K. N., Nishu Singla, Deepak Kumar Singhal, Ritesh Singla

**Affiliations:** ^1^ Department of Public Health Dentistry Manipal College of Dental Sciences Manipal Academy of Higher Education, Manipal, Udupi, Karnataka, India; ^2^ Department of General Medicine Kasturba Medical College Manipal Academy of Higher Education, Manipal, Udupi, Karnataka, India; ^3^ Department of Orthodontics and Dentofacial Orthopaedics Manipal College of Dental Sciences Manipal Academy of Higher Education, Manipal, Udupi, Karnataka, India

## Abstract

**Objectives:**

To compare the prevalence of root surface carious lesions among the nondiabetic and diabetic elderly population and its association with various risk factors.

**Methods:**

An observational analytical cross-sectional study was conducted among 800 elderly participants, 431 males and 369 females, aged 50 years and above, with a minimum of ten teeth present, with 400 being diabetic and 400 nondiabetic from the various hospitals of the Udupi district. Demographics, socioeconomic status, oral hygiene practices, oral abusive habits, and history of type 2 diabetes were collected using an interviewer-administered questionnaire followed by an intraoral examination to assess root caries. The primary outcome measure was the presence of any carious or filled root surface. Binary logistic regression univariate analysis was done for all the predictor variables, and those with significant associations were further analyzed by multivariate analysis with the enter method in a single-step model. An appropriate nomogram was designed for risk prediction.

**Results:**

Overall, the root caries prevalence was 37.3%, and the mean root caries index was 14.28%. The prevalence of root caries was 46% in people with diabetes and 28.5% in nondiabetics. Participants with type 2 diabetes, less education, low socioeconomic status, and using finger cleaning had a significantly higher risk of developing root caries. Additionally, those who used removable partial dentures (RPDs) were found to be 4.65 times more likely to have root caries than those who did not use RPDs.

**Conclusion:**

Elderly diabetics are at a higher risk for developing root caries and are strongly advised to maintain good oral hygiene practices and to undergo periodic dental evaluations. Therefore, it is crucial to emphasize early diagnosis and treatment of root caries in this population.

## 1. Introduction

The aging population is a phenomenon seen worldwide. In developing countries like India, the aged population is expanding rapidly. The fraction of the population aged 60 years and above was 7 percent in 2009 (88 million) and is estimated to increase to 20 percent (315 million) by the year 2050. Although the proportion of India's population aged 50 and above is currently relatively small at 16 percent, India is projected to experience rapid growth among this age group in the coming decades. The Population Division of the United Nations has predicted that the population above 50 years in India will reach 34 percent by 2050 [1].

The proportion of older people has increased due to an overall increase in life expectancy and improved availability of healthcare resources. As people age, their dental and overall health becomes an increasingly significant concern [2]. As individuals get older, oral health concerns can manifest in different ways. Some may experience tooth loss and become edentulous, while others may retain their teeth but encounter issues such as periodontitis, xerostomia, and root caries [3].

Root caries is one of the significant oral health problems in the elderly. Root surface caries is a bacterial-induced carious lesion on the root surface of a tooth with gingival recession [4, 5]. As people age, they become more susceptible to root caries due to the cumulative effects of long-term neglected oral hygiene and harmful habits such as smoking, tobacco chewing, increased bone resorption, gingival recession, dry mouth, and other systemic factors [6]. Systemic conditions such as diabetes mellitus can lead to oral health problems such as periodontitis as well as dental caries.

The relationship between diabetes and periodontitis is established and is known to impact each other significantly [7]. Periodontal complication is the sixth complication of diabetes mellitus. Elderly individuals with diabetes have a greater risk of developing root caries due to potential periodontal complications [8]. Those with type 2 diabetes who experience a higher incidence of root caries may be more susceptible to increased levels of glucose in their oral secretions. This can lead to a more significant growth of anaerobic microflora in the gingival sulci, promoting aciduric and acidogenic bacteria related to root surfaces or coronal caries [9, 10].

In the past two decades, many authors worldwide have reported varied occurrences of root caries among older individuals between 9 and 89%, mostly between 30 and 60% [11–13]. Several studies conducted in India have reported a high occurrence of root caries in elderly individuals, with prevalence ranging from 41.9% to 46.4% [14–16]. A meta-analysis conducted by Pentapati et al., which included 74 publications, found a pooled prevalence of 41.5% (with a range of 36.9% to 46.1%) for root caries [6]. Only two out of 74 publications reported the prevalence of root caries among diabetic patients, and most studies did not specify comorbid conditions and corresponding prevalence [10, 17]. However, in the South Indian population, there are minimal studies regarding the root caries prevalence among diabetic elderly populations.

Hence, our study aimed to assess the prevalence of root carious lesions among diabetic and nondiabetic elderly people and to find their relation to various related risk factors. It was hypothesized that people with diabetes, owing to their systemic condition, will have a higher root caries prevalence as compared to nondiabetics.

## 2. Methods

An observational analytical cross-sectional study was conducted between January 2015 and September 2016 among the elderly population visiting the major hospitals in all three taluks of Udupi district, Karnataka state. The approval to perform the study was obtained from the Institutional Ethics Committee. After obtaining approval from the ethical committee, further permission was obtained from the respective Chief Medical Officers of the various hospitals to conduct the study.

A pilot study was carried out on 50 participants (25 diabetics, 25 nondiabetic) to estimate the prevalence of root caries among both groups. The root caries prevalence obtained from the pilot study was 30% in the nondiabetic population and 40% in the diabetic population. Hence, a clinically significant difference of 10% was considered between the two groups, with a 5% level of significance and 80% power of the study. Calculation R package version 3.3.1 was used for sample size calculation. A sample size of 356 patients, approximately 400 in each of the two groups, was found to be appropriate for the study. The pilot study participants were not considered for the main study results.

Inclusion criteria were participants aged 50 years and above with a minimum of ten teeth present with type 2 diabetes mellitus for the diabetic population and without type 2 diabetes mellitus for the nondiabetic population. Participants who were not aware of their current diabetic status and subjects with severe comorbidities limiting recording of their oral health status were excluded.

Based on the population distribution in the three taluks of Udupi district, a representative population was taken from each taluk. 50% of the participants were taken from Udupi, 30% from Kundapura, and 20% from Karkala Taluk [18].

Further, in the Udupi taluk, one government and two private hospitals were selected randomly. In Kundapura and Karkala, one government hospital and one private hospital each were chosen randomly. A non-probability-based purposeful quota sampling procedure was followed to select respondents. Subjects reporting to the hospital were first given information about their participation in the study. Those who were willing to participate signed a written informed consent.

Data on basic demographics, socioeconomic status, oral hygiene practices, oral abusive habits, and history of type 2 diabetes were collected using a standard format. Socioeconomic status was calculated per the modification of BG Prasad's classification, which was based on the participants' per capita monthly income and applicable to rural and urban areas [19].

Before the start of the study, training and calibration exercises were performed by the principal investigator. The training session was conducted under the guidance of a senior examiner who is well versed in recording root caries. Interexaminer kappa value was 0.78. The senior examiner also trained a recorder to record the findings accurately.

A face-to-face personal interview method was used to obtain complete information from the respondents. Participants were initially asked about their history of diabetes, and those who confirmed were recorded as diabetic and others as nondiabetic. The results were further confirmed by their blood sugar values from hospital records. Participants uncertain about their diabetic status were excluded.

The intraoral examination included recording coronal caries experience (DMFT) and root caries status. For each tooth, the presence of coronal caries was assessed by the WHO criteria [20]. Root surface caries was formulated as the Root Caries Index (RCI) proposed by Katz in 1986, scoring root surfaces as sound, carious, or filled [21]. RCI is the proportion of visible/exposed susceptible root surfaces with caries (decayed and/or filled surfaces) [22].

RCI was computed as follows:

(1) $$\begin{array}{c} \text{Root Caries Index }\left(\text{RCI}\right)=\left\{\frac{\left(R-D\right)+\left(R-F\right)}{\left(R-D\right)+\left(R-F\right)+\left(R-N\right)}\right\}\times 100. \end{array}$$

Recession with decayed root surface (R–D); recession with filled root surface (R–F); recession with sound root surface (R–N).

Four exposed root surfaces were recorded for each included tooth, and the root surfaces without recession were not recorded.

Examinations were performed on a dental chair with a light source or on a regular chair under a natural/external light source as per availability. All the necessary infection control procedures were followed during the examination.

Data collected through a standard form and clinical examination were entered into an Excel sheet. The data were analyzed using IBM-SPSS software ver: 15.0 (Chicago, IL) and R Package ver. for statistical analysis. The independent variables considered in the study were age, gender, area of residence, marital status, education, socioeconomic status, oral hygiene practices (including method, frequency, and material used for cleaning), oral abusive habits (such as smoking, smokeless tobacco use, and alcohol consumption), use of removable partial dentures, history of type 2 diabetes, duration of diabetes, and DMFT status as low and high (decayed, missing, and filled teeth). The primary outcome or the dependent variable was dichotomized as the absence or presence of root caries lesions (filled or decayed).

Logistic regression analysis was performed to evaluate the relationship between various predictor variables (either categorical or continuous) and an outcome that is binary or dichotomous. Binary logistic regression univariate analysis was performed to find the odds ratio and the significant relationship of the outcome with each predictor, one at a time. All the independent variables with a significance level of 0.05 or less were considered for multivariate logistic regression to obtain a confounder-adjusted odds ratio. In the “Enter” method (the default option on many statistical programs), all the input variables are entered simultaneously. This means that all the independent variables will be given equal importance in the model, from which some may remain significant and some become insignificant. The Hosmer–Lemeshow test was selected in SPSS while performing logistic regression analysis and found a good fit for the data (*p*=0.315). A *p* value of <0.05 was considered as the level of significance.

The significant variables were used to build a nomogram using the R package software. A nomogram is a predictive tool. This creates a simple graphical representation of a statistical predictive model that generates a numerical probability of a clinical event [23].

## 3. Results

The study involved 800 participants, consisting of 431 (53.8%) males and 369 (46.2%) females, with an equal ratio of 400 diabetic and 400 nondiabetic subjects. The participants were divided into three age groups, with 209 (26.1%) being between 50 and 60 years, 358 (44.7%) between 61 and 70 years, and 233 (29.1%) between 71 and 80 years. The study population consisted of 495 (61.8%) individuals from rural areas, with 100 (12.5%) from the upper socioeconomic class, 285 (35.62%) from the middle class, and 415 (51.8%) from the lower middle and lower socioeconomic classes. A total of 82 (10.2%) participants were illiterate, 379 (47.4%) had education till primary/middle school, 100 (12.5%) till high school, and 239 (29.8%) had a diploma or higher education. Of the participants, 622 (77.8%) were married and 178 (22.3%) were unmarried or categorized as “others.” In terms of diet, 410 (51.2%) participants consumed a vegetarian diet, while 390 (48.8%) had a mixed diet.

According to this study, 727 people (90.9%) used toothbrushes to clean their teeth, while 73 (9.1%) used their fingers. Of these, 744 individuals (93%) used toothpaste, whereas 58 (7%) used toothpowder. Additionally, 635 people (79.4%) cleaned their teeth once daily, whereas the remaining 165 (20.6%) cleaned them more than once daily. Moreover, 238 people (29.7%) had the habit of smoking, 208 (26%) had the habit of alcohol consumption, and 279 (34.9%) consumed smokeless tobacco. The study also found that 204 people (25.5%) used removable partial dentures.

The mean score of the number of teeth with recession and sound root surfaces (R–N) was 10.24 ± 7.63, with a median of 9.00 and a range of 0–47. The mean score for the number of teeth having recession and decayed root surfaces (R-D) was 1.71 ± 2.98 (range 0–16). The median of coronal DMFT was 4 (range 0–18). There were no root surfaces with any filling present. This population's mean root caries index (RCI) was 14.28%.

(2) $$\begin{array}{c} \text{Root Caries Index }\left(\text{RCI}\right)=\left\{\frac{\left(R-D\right)+\left(R-F\right)}{\left(R-D\right)+\left(R-\;F\right)+\left(R-N\right)}\right\}\times 100,\\ \text{RCI}=\left\{\frac{\left(1365\right)+\left(0\right)}{\left(1365\right)+\left(0\right)+\left(8193\right)}\right\}\times 100=\frac{1365}{9558}=14.28\%. \end{array}$$

Overall, the prevalence of root caries was 37.3%. Participants with type 2 diabetes had two times higher likelihood of developing root caries than nondiabetics with an odds ratio of 1.97 (1.38–2.81) (*p* < 0.001).

The taluk-wise distribution did not show much variation in the prevalence of root caries as Udupi taluk had 36%, Kundapura taluk 40%, and Karkala taluk 36.3%, respectively.

Table 1 depicts the study population distribution per the root caries prevalence and diabetes status, DMFT, and root surfaces with recession. Root caries was significantly more common among people with diabetes in 184 (46%) of the diabetic population and 114 (28.5%) of the nondiabetic population (*p* < 0.001). Additionally, root caries was found to have a significantly increased association with the increase in root surfaces with recession. As the variable “a number of root surfaces with recession” is implicit in the dependent variable, it was removed from the multivariate model to avoid the issue of mathematical coupling.

Table 2 depicts the study population distribution as per the root caries prevalence and sociodemographic characteristics. It was found that participants living in rural areas (*p*=0.02), consuming a mixed diet (*p*=0.02), having primary education (*p*=0.002), and having a low socioeconomic status (*p* < 0.001) were significantly more likely to develop root caries.

Table 3 depicts the study population distribution as per the root caries prevalence, adverse habits, and oral hygiene practices. It was observed that the participants using fingers as a method of cleaning (*p*=0.001) and using toothpowder to clean teeth (*p*=0.02), had the habit of smokeless tobacco chewing (*p*=0.05), and using Removable Partial Dentures (RPD) (*p* < 0.001) were significantly more likely to have root caries.

Table 4 depicts multiple logistic regression outcomes for the prevalence of root caries. It was found that people who had completed primary or high school education were nearly twice as likely to develop root caries compared to those who had a diploma and higher degree, with an adjusted odds ratio of 1.94 (*p*=0.004) and 1.61 (1.01–2.55) (*p*=0.04), respectively. Individuals with lower socioeconomic status had significantly higher odds of 2.19 (1.40–3.42) (*p*=0.001) of having root caries, while middle-class individuals had a 1.67 (1.09–2.56) (*p*=0.01) odds ratio as compared to upper-class people. Also, using fingers to clean teeth increases the risk of developing root caries significantly, with an odds ratio of 2.05 (1.09–3.86) (*p*=0.02). Additionally, those who used Removable Partial Dentures (RPDs) were found to be 4.65 (3.10–6.88) times more likely to have root caries compared to those who did not use RPDs (*p* < 0.001).

**Figure 1:**
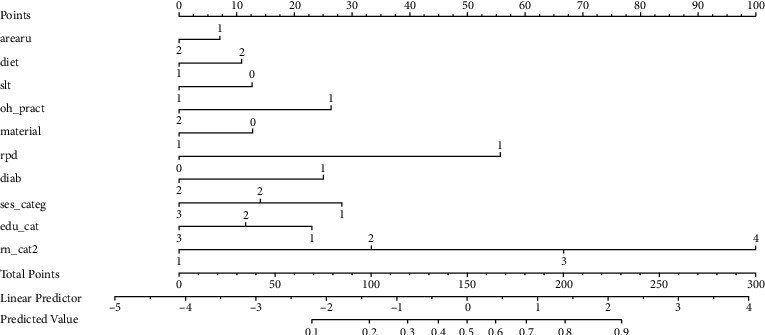
Nomogram showing root caries risk prediction.

Figure 1 shows the nomogram for root caries risk prediction. Supporting information on using nomograms is also available.

Refer to supporting information for variable coding and details on using nomograms. How to use a nomogram: each subtype within the variables is assigned a score. An individual patient's value is located on each variable axis, and a line is drawn upward to determine the number of points received for each variable value. By adding up the total score from all the variables and locating it to the total point scale, we could determine the probabilities of the outcomes by drawing a vertical line to the total score. The coding for various variables is as follows:

- arearu is an area of residence coded as (1-rural, 2-urban).
- diet coded as (1-veg, 2-mixed).
- slt is use of smokeless tobacco coded as (0-no use of smokeless tobacco, 1-uses smokeless tobacco).
- oh_pract is oral hygiene practice coded as (1-uses finger to clean, 2-uses a toothbrush to clean).
- material is material used to clean teeth coded as (0-toothpowder, 1-toothpaste).
- rpd is use of rpd coded as (0-no use of rpd, 1-use of rpd).
- diab is the history of diabetes coded as (1-history of diabetes, 2-no history of diabetes).
- ses_categ is the socioeconomic status coded as (1-low, 2-medium, 3-high).
- edu_cat is the level of education status coded as (1-primary, 2-high school, 3-diploma, and above).
- rn_cat2 is tooth surfaces with recession coded as (1-0–4 surfaces, 2-5–9 surfaces, 3-10–14 surfaces, and 4-15 and above surfaces).

## 4. Discussion

The present study was conducted to find root surface carious lesions' prevalence and assess the associated risk factors among elderly diabetic and nondiabetic people in Udupi district, Karnataka. Diabetes mellitus (DM) is a modern-day disease affecting more than 62 million individuals in India. India ranks among the top 3 countries with a diabetic population [24]. This study showed that the root caries prevalence was significantly higher among diabetics (46%) than among nondiabetics (28.5%). The findings of the current study confirmed as per the study conducted in Bangalore, India, by Soni et al., which reported a prevalence of 42% among diabetics [25]. The results of our research also confirmed with the study conducted in Thailand by Hintao et al., who reported a prevalence of root caries of 40% among diabetics and 18.5% among nondiabetic subjects. [10].

The overall prevalence of root surface carious lesions in our study participants was 37.3%, slightly less than that of the prevalence (46.4%) reported by Kumara and Radha et al. in India [14]. This confirms with the study by Imazato et al. in Japan [12]. However, authors from Sri Lanka [11] have reported a relatively greater prevalence of root carious lesions in the elderly population at 89.7%, and authors from Brazil [26] have reported a prevalence of 78%. The mean RCI was 14.28% in our study population, which was similar to findings obtained in studies from India [14], Brazil [26], and Thailand [15].

In addition to evaluating the prevalence of root caries, this study also examined the risk factors associated with its occurrence. The multivariate model showed the status of type 2 diabetes, the level of education, socioeconomic status, the method of cleaning teeth, use of RPD, and the number of teeth having recession as the statistically significant determinants of root carious lesions. It is important to note that no restoration was observed on any root surface in our study population. The findings of this study suggest that the participants had limited knowledge and usage of dental services when it comes to restoring root caries. Moreover, the elderly individuals in the study population might have encountered difficulties accessing nearby dental healthcare services.

It was also found that people living in rural areas (40.2%) had a significantly higher prevalence of root caries than those living in urban areas (32.5%). In a related study conducted in the same region, urban residents exhibited better oral hygiene and gingival status and less periodontitis than rural participants [27]. This could be attributed to various factors, such as differences in education, occupation, income, lifestyle, and access to healthcare services between urban and rural populations [27].

The linkage between socioeconomic status and health, including oral health, is well established [28]. In this study, people with lower socioeconomic status had twice the likelihood of having root caries than those with higher socioeconomic status. The most important indicators of socioeconomic status (SES) are occupational status, income, and level of education. In our study, occupation was not considered a predictor because most respondents in this age group were retired and were either dependent on pension or the income of other family members. Those participants with only primary school education had a significantly higher association with root caries than those with higher education.

Many other variables influence the initiation and progression of carious lesions; however, one of the most critical variables is oral hygiene, as efficient oral hygiene practices prevent the formation of plaque on tooth surfaces [29]. Our study also found that using a toothbrush as a cleaning method had a protective effect on root caries. However, removable partial dentures attribute greater odds to root surface carious lesions owing to the position of clasp placement. The clasps attached to conventional RPDs are in close approximation with root surfaces. They make a favorable environment for plaque accumulation and further bacterial invasion, eventually leading to root caries [30]. This finding was supported in our study. The participants who were using any form of RPD had a significantly increased risk of getting root caries as compared to nonusers.

It is widely recognized that smoking is a significant risk factor for dental caries [31]. But, in this population, participants smoking tobacco had a slightly lower risk of having root caries as compared to those who were not smoking. However, the results were not statistically significant. This contrary finding is in line with Hintao et al., where current and ex-smoking were significantly negatively associated with coronal caries. The reason for this needs to be further explored in greater detail. Still, a probable explanation could be that the fewer participants in the smoking group could have led to overestimating the results.

Very few studies have evaluated the association between root carious lesions and the habit of smokeless tobacco chewing [32]. Studies reported have indicated a negative effect of tobacco use on oral health. However, the results of this study showed that individuals consuming smokeless tobacco had a lower risk of developing root caries. Tobacco or betel chewers may have lesser carious lesions because extrinsic tobacco stains on tooth/root surfaces may be protective against bacterial acid release by acting as a chemical or physical barrier [11]. Staining tobacco teeth also limits the proper recording of root caries in individuals. In our study population, participants with a mixed diet had a significantly greater association with root caries. A possible reason for this could be the presence of cariostatic tannins and phytins in green vegetables [33].

There was no association between root caries and total DMFT score. However, other authors have reported an association between the presence of coronal carious lesions and root caries [34]. When assessed as a risk factor, the number of recession surfaces showed that the root caries increased exponentially with an increasing number of surfaces. Individuals with 15 and above exposed root surfaces had fifteen times the risk of developing root caries, which shows that a recession surface is the highest predictor of root caries. Similar results were reported in other studies [35].

The nomogram model showed the relative score of the significant predictors on a graduated scale. The risk prediction of any individual can be calculated by adding the relative score and getting the overall risk from 0 to 100. It provides an insight into the weight of each variable. The variable that has the most impact on the outcome is given a score of 100 points. The other variables are then given a score based on the magnitude of their impact relative to the most important variable. Comparing the score of the most significant variable to the least significant variable can help identify their relative importance (Figure 1).

### 4.1. Limitations

This study has a few limitations that need to be taken into consideration. Firstly, the sample used in the study was taken from a hospital-based population alone, which may not accurately represent the entire population. Furthermore, this study did not consider various predictors, such as plaque, xerostomia, fluoride intake, stress levels, salivary pH, and other systemic diseases. Lastly, it is worth noting that the diagnosis of root caries was based solely on traditional visual-tactile methods, and no radiographs were used. Due to these limitations, the interpretation of study results should be cautious.

### 4.2. Conclusion

In conclusion, the diabetic elderly population had significantly greater root caries than the nondiabetic population. The various risk factors associated with root caries were the tooth surfaces with recession, usage of Removable Partial Dentures, socioeconomic status, the level of education, and the method of cleaning teeth. Elderly diabetics are at a higher risk for developing root caries and are strongly advised to maintain good oral hygiene practices and to undergo periodic dental evaluations. Therefore, it is crucial to emphasize early diagnosis and prompt treatment of root caries in this population.

### 4.3. Recommendations

As a learning from this study, it is advised to screen all geriatric populations for root caries in particular with a special focus on root caries and also provide appropriate treatment for the same. However, as a follow-up of this research, it is recommended to conduct long-term studies exploring the risk factors that could determine the causal relationship of root caries.

## Figures and Tables

**Table 1:** Distribution of the study subjects according to the prevalence of root caries and diabetes history, past duration of diabetes, DMFT status, and tooth surfaces with recession (*n* = 800).

Variable (*n*)	Root caries	OR (95% CI)	*p* value
History of type II DM			
Yes (400)	184 (46%)	2.13 (1.59–2.86)	<0.001
No (400)	114 (28.5%)	1	
Past duration of diabetes			
<5 years (132)	60 (45.5%)	1	—
5–10 years (140)	69 (49.3%)	1.16 (0.72–1.19)	0.52
>10 years (128)	55 (43.0%)	0.90 (0.55–1.47)	0.68
DMFT^*∗*^			
Low (323)	119 (36.8%)	1	
High (477)	179 (37.5%)	1.03 (0.77–1.38)	0.84
Tooth surfaces with recession^#^			
0–4 (194)	28 (14.4%)	1	—
5–9 (218)	57 (26.1%)	2.10 (1.27–3.47)	0.004
10–14 (186)	68 (36.6%)	3.41 (2.07–5.63)	<0.001
15 and above (202)	145 (71.8%)	15.08 (9.11–24.97)	<0.001

*p* < 0.05 statistically significant. ^*∗*^DMFT < 4 as low and DMFT 5 and above as high based on the median score. ^#^Surfaces were categorized based on the cut-off obtained for the median (25th, 75th percentile).

**Table 2:** Distribution of the study subjects according to the prevalence of root caries and sociodemographic characteristics (*n* = 800).

Variable (*n*)	Root caries	OR (95% CI)	*p* value
Area of residence			
Rural (495)	199 (40.2%)	1.40 (1.03–1.89)	0.02
Urban (305)	99 (32.5%)	1	
Age (in years)			
50–60 (209)	71 (34.0%)	1	—
61–70 (358)	136 (38.0%)	1.19 (0.83–1.70)	0.34
71–80 (233)	91 (39.1%)	1.24 (0.84–1.83)	0.27
Gender			
Male (431)	159 (36.9%)	1.03 (0.78–1.38)	0.82
Female (369)	139 (37.7%)	1	
Diet			
Vegetarian (410)	137 (33.4%)	1	
Mixed (390)	161 (41.3%)	1.40 (1.05–1.86)	0.02
Marital status			
Married (622)	228 (36.7%)	1	—
Unmarried (152)	61 (40.1%)	1.15 (0.80–1.66)	0.42
Others^*∗*^ (26)	9 (34.6%)	0.91 (0.40–2.08)	0.83
Education			
Primary (291)	126 (43.3%)	1.78 (1.23–2.54)	0.002
High school (270)	100 (37.0%)	1.36 (0.94–1.98)	0.10
Diploma and above (239)	72 (30.1%)	1	—
Socioeconomic status			
Upper (258)	72 (27.9%)	1	—
Middle (307)	114 (37.1%)	1.52 (1.06–2.18)	0.02
Low (235)	112 (52.3%)	2.35 (1.62–3.42)	<0.001

*p* < 0.05 statistically significant. ^*∗*^includes widows and divorcees.

**Table 3:** Distribution of the study subjects according to the prevalence of root caries and oral hygiene practices and adverse habits (*n* = 800).

Variable (*n*)	Root caries	OR (95% CI)	*p* value
Method of cleaning teeth			
Toothbrush (727)	258 (35.5%)	1	
Finger (73)	40 (54.8%)	2.20 (1.36–3.58)	0.001
Material used for cleaning			
Toothpaste (744)	269 (36.2%)	1	
Toothpowder (56)	29 (53.5%)	1.89 (1.10–3.27)	0.02
Frequency of cleaning			
Once a day (635)	237 (37.3%)	1.01 (0.71–1.44)	0.93
More than once (165)	61 (37.0%)	1	
Use of RPD			
Yes (204)	124 (60.8%)	3.76 (2.69–5.23)	<0.001
No (596)	174 (29.2%)	1	
Smoking			
Yes (238)	80 (33.6%)	0.80 (0.58–1.09)	0.16
No (562)	218 (38.8%)	1	
Smokeless tobacco			
Yes (279)	91 (32.6%)	0.73 (0.54–1.0)	0.04
No (521)	207 (39.7%)	1	

*p* < 0.05 statistically significant.

**Table 4:** Multiple logistic regression for root caries presence.

Independent variable	Categories	Adjusted OR (95% CI)	*p* value
Area of residence	Rural	1.18 (0.82–1.71)	0.38
	Urban	1	

Diet	Mixed	1.29 (0.90–1.83)	0.16
	Vegetarian	1	

Education	Primary	1.94 (1.24–3.03)	0.004
	High school	1.61 (1.01–2.55)	0.04
	Diploma and above	1	—

Socio economic status	Low	2.19 (1.40–3.42)	0.001
	Middle	1.67 (1.09–2.56)	0.01
	Upper	1	—

Method of cleaning teeth	Finger	2.05 (1.09–3.86)	0.02
	Toothbrush	1	

Material used for cleaning	Toothpowder	1.38 (0.67–2.80)	0.37
	Toothpaste	1	

Use of RPD	Yes	4.62 (3.10–6.88)	<0.001
	No	1	

Smokeless tobacco	Yes	0.71 (0.49–1.03)	0.07
	No	1	

History of type II diabetes	Yes	1.97 (1.38–2.81)	<0.001
	No	1	

*p* < 0.05 statistically significant.

## Data Availability

The data that support the findings of this study are available on reasonable request to the corresponding author.
